# Changes in Optical Properties of Plasmonic Nanoparticles in Cellular Environments are Modulated by Nanoparticle PEGylation and Serum Conditions

**DOI:** 10.1186/s11671-016-1524-4

**Published:** 2016-06-18

**Authors:** Allen L. Chen, Meredith A. Jackson, Adam Y. Lin, Elizabeth R. Figueroa, Ying S. Hu, Emily R. Evans, Vishwaratn Asthana, Joseph K. Young, Rebekah A. Drezek

**Affiliations:** Department of Bioengineering, Rice University, Houston, 77005 TX USA; Waitt Advanced Biophotonics Center, Salk Institute for Biological Studies, La Jolla, 92037 CA USA; Department of Electrical and Computer Engineering, Rice University, Houston, 77005 TX USA

**Keywords:** Nano-bio interactions, Plasmonics, Hyperspectral imaging, Gold nanoparticles, Cells, Spectral shifting, Nanomedicine, Poly(ethylene glycol), Serum, Protein corona

## Abstract

When plasmonic nanoparticles (NPs) are internalized by cells and agglomerate within intracellular vesicles, their optical spectra can shift and broaden as a result of plasmonic coupling of NPs in close proximity to one another. For such optical changes to be accounted for in the design of plasmonic NPs for light-based biomedical applications, quantitative design relationships between designable factors and spectral shifts need to be established. Here we begin building such a framework by investigating how functionalization of gold NPs (AuNPs) with biocompatible poly(ethylene) glycol (PEG), and the serum conditions in which the NPs are introduced to cells impact the optical changes exhibited by NPs in a cellular context. Utilizing darkfield hyperspectral imaging, we find that PEGylation decreases the spectral shifting and spectral broadening experienced by 100 nm AuNPs following uptake by Sk-Br-3 cells, but up to a 33 ± 12 nm shift in the spectral peak wavelength can still occur. The serum protein-containing biological medium also modulates the spectral changes experienced by cell-exposed NPs through the formation of a protein corona on the surface of NPs that mediates NP interactions with cells: PEGylated AuNPs exposed to cells in serum-free conditions experience greater spectral shifts than in serum-containing environments. Moreover, increased concentrations of serum (10, 25, or 50 %) result in the formation of smaller intracellular NP clusters and correspondingly reduced spectral shifts after 5 and 10 h NP-cell exposure. However, after 24 h, NP cluster size and spectral shifts are comparable and become independent of serum concentration. By elucidating the impact of PEGylation and serum concentration on the spectral changes experienced by plasmonic NPs in cells, this study provides a foundation for the optical engineering of plasmonic NPs for use in biomedical environments.

**Electronic supplementary material:**

The online version of this article (doi:10.1186/s11671-016-1524-4) contains supplementary material, which is available to authorized users.

## Background

Plasmonic metal nanoparticles (NPs) are employed in a variety of biomedical applications, ranging from in vitro quantitative study of biological processes to in vitro and in vivo photonic gene circuits and photothermal therapy [1–7]. Optical excitation of metal NPs at particular wavelengths generates a resonant collective oscillation of the NPs’ conduction electrons, termed “plasmon resonance,” which manifests as strong absorption or scattering of light. The wavelength of light at which metal NPs exhibit maximal plasmon resonance is highly sensitive to the NPs’ geometry, compositional material, and local environment [8]. This relationship enables metal NPs to be engineered for optical absorption or scattering at desired wavelengths for biomedical applications by fabricating NPs with a geometry and material composition that provide the desired optical response. Metal NPs designed in this fashion have been developed for use as scattering contrast markers [1, 2], optical heat transducers [7, 9], and multiplexed light-activated gene-delivery agents [3, 10]. However, it has been shown that the cellular uptake and agglomeration of plasmonic nanoparticles in biological environments can result in coupling of optical resonances, leading to a shift in the optical spectra of metal nanoparticles [11–13].

Recent advancements in darkfield imaging and processing have enabled spectra of plasmonic nanoparticles in cells to be captured more efficiently [14–17], therefore making it possible to quantify the extent of these spectral shifts. We recently developed a darkfield hyperspectral (HS) imaging and analysis approach to systematically quantify the magnitude of these spectral shifts, the associated spectral broadening, and the variability of the spectral shifts among cells in a cell population using 100-nm bare gold NPs (AuNPs) as a model NP [13]. These results provided quantitative metrics for the first time for assessing the significance of the spectral shifts that occur in a cellular environment. In order for NPs to be more precisely designed to account for such cell-associated spectral changes in biomedical applications (preventing unintended optical effects and achieving more precise, predicted optical response), there is a need for further understanding of how NP design parameters or environmental factors can influence the spectral changes that are realized in a cellular environment. For example, it is unknown how NP size, surface chemistry, or cell type will impact the magnitude of spectral change or broadening of the NP spectra in a cellular environment. To build a framework for understanding how much designable parameters will influence the resulting spectral changes—which can aid in accounting for the changes differently based on the parameters used in an application or to aid in choosing the proper parameters during NP design to elicit or avoid the spectral shift—here we study the effect of (1) coating NPs with poly(ethylene glycol) (PEG) and (2) serum concentration in the cellular environment.

PEG is one of the most popular coatings for NPs to be used in in vivo applications because its hydrophilic and steric nature minimizes protein adsorption and reduces detection by the reticulo-endothelial clearance system (RES), prolonging blood circulation for improved cellular target delivery [18]. In certain in vitro cellular diagnostic or gene-delivery applications, PEG-free NPs are used [3, 5, 19] while in other applications, especially those that are in vivo, NPs are often functionalized with PEG (“PEGylated”) [20, 21]. Walkey and colleagues demonstrated that AuNPs not grafted with PEG (“bare AuNPs”) or grafted with different densities of PEG exhibited major differences in the cellular uptake and intracellular agglomeration state [22]. Transmission electron microscopy (TEM) imaging showed that increasing the PEG density decreased intracellular agglomeration of NPs and reduced the number of NPs per vesicle following incubation with J774A.1 macrophages [22]. These previous results suggest a possibility that the extent of spectral shifting for AuNPs following cellular interaction could depend on whether they are PEGylated since the optical properties of plasmonic NPs are highly sensitive to their local dielectric environment and proximity to other plasmonic NPs [8, 23, 24]. Furthermore, it is unknown if the added PEG layer on PEGylated AuNPs provides a sufficient separation distance between NPs when PEGylated AuNPs are clustered within intracellular vesicles, which is capable of decreasing plasmonic coupling-associated spectral shifts. Because NPs in many applications are PEGylated, it is therefore important to understand how this factor affects the extent of the shift in the NP spectra that occurs. In this paper, we seek to quantitatively assess the shifts that occur when a NP is PEGylated compared with when it is bare to allow for finer design of the NP optical response for a given application.

In addition to the properties of the NP, the role of the biological environment in influencing the extent of these shifts also needs to be considered. Plasmonic NPs are being utilized in applications spanning from serum-free to serum-containing conditions of various serum concentrations [5, 25–29]. Given the importance of the NP’s protein corona in mediating interactions between NPs and cells (e.g., cellular uptake and subsequent intracellular transport) [30–33], we hypothesized that the presence and absence of serum proteins during NP-cell interaction may importantly modulate the intracellular agglomeration and spectral shift accompanied by nanoparticles. Lesniak and Dawson previously showed that silica NPs incubated with A549 cells in serum-free media had higher uptake and distinct intracellular localization compared with when incubated with cells in 10 % serum-containing culture media due to different mechanisms of interaction with cells [34]. Zhu and Rotello also showed that increased serum concentration (from 0 to 10 to 50 % serum) significantly decreased uptake of 2-nm AuNPs in HeLa cells [35]. Dawson et al. also recently showed that NP-cell interactions differ remarkably in 10 % (in vitro) and 100 % (in vivo mimicking) serum concentration conditions [29]. These previous studies led us to hypothesize that increased serum concentrations and a move from in vitro to in vivo serum levels may lead to smaller NP agglomerates within cells and smaller shifts in spectra. Such differences could have implications in the relative importance of considering cellular uptake-associated spectral changes of NPs in in vitro applications compared with in in vivo applications. Consequently, we study the effect of serum concentration on shifts of the spectra of PEGylated AuNPs following cellular uptake, aiming to determine to what extent the NP spectra may additionally change in conditions when the serum concentration may be different.

By applying the HS imaging and analysis approach we previously established, in this paper, we therefore quantify the spectral changes experienced in cellular environments by PEGylated AuNPs as compared with bare AuNPs and study how spectra are further impacted by serum concentration (0, 10, 25, or 50 % human serum). We explain the impact of these NP design and environmental factors on the spectral changes exhibited by considering the differences in NP protein corona and intracellular agglomeration. This study demonstrates how HS imaging can be utilized to construct a design framework to enable more precise design of plasmonic NPs for light-based biomedical applications.

## Methods

### Functionalization of AuNPs with PEG

Citrate-stabilized spherical AuNPs (100 nm, Ted Pella) were sonicated to ensure particles were well-separated before functionalization. To functionalize AuNPs with PEG, AuNPs at a concentration of 5.6 × 10^9^ NPs/mL were incubated overnight with 11.6 μM (end concentration) of methoxy-terminated thiolated PEG (mPEG-SH, MW = 5000 Da, NanoCS). To ensure optimal surface coverage of PEG, the solution was progressively raised to 10-mM sodium phosphate, 0.1 % *v*/*v* Tween 20, and 0.1 M NaCl and incubated overnight. Excess PEG molecules were then removed by three washing steps of centrifugation at 3000*g* for 30 min followed by re-suspension in milliQ water. PEGylated AuNPs were stored at 4 °C until use.

### Introducing PEGylated AuNPs to Cells

Sk-Br-3 breast adenocarcinoma cells (American Type Culture Collection) were cultured in McCoy’s 5A growth media supplemented with 10 % *v*/*v* human off-the-clot type AB serum (Valley Biomedical) and 1 % penicillin-streptomycin and maintained at 37 °C in a 5 % CO_2_ incubator. For incubation time and exposure dose experiments, Sk-Br-3 cells were plated in LabTek II CC2 four-well chamber slides at a density of 100,000 cells/mL and grown to 70 % confluence. After 24 h, culture media was removed and cells were incubated with PEGylated AuNPs in complete phenol-red free media (CPRFM) containing 10 % HuS for 2, 5, 10, or 24 h in an incubator at 37 °C and 5 % CO_2_ as described previously in detail [13]. For human serum (HuS) concentration experiments, Sk-Br-3 cells were cultured in McCoy’s 5A media supplemented with 1 % penicillin-streptomycin and either 10, 25, or 50 % *v*/*v* human off-the-clot type AB serum (Valley Biomedical). Sk-Br-3 cells cultured in media containing 25 or 50 % *v*/*v* HuS were weaned progressively from media containing 10 % *v*/*v* HuS through the course of multiple passages. Cells were then plated into LabTek II CC2 four-well chamber slides at a density of 100,000 cells/mL in media containing their respective concentration of serum. After 24 h, culture media was removed from wells. Wells with cells cultured in media with 10 % *v*/*v* HuS were then incubated with 24 μg/mL of PEGylated AuNPs either in PRFM (0 % HuS) or in CPRFM containing 10 % HuS. Wells with cells cultured in media with 25 % or 50 % *v*/*v* HuS were incubated with 24 μg/mL PEGylated AuNPs in CPRFM containing their respective HuS concentrations. Cells were incubated with PEGylated AuNPs in these serum concentration conditions (0, 10, 25, or 50 % HuS) for 5, 10, or 24 h before spectral measurements were performed.

### Measuring Optical Spectra for PEGylated AuNPs Introduced to Cells

Following incubation with PEGylated AuNPs, cells were rinsed three times with 1X Dulbecco’s phosphate buffered saline without magnesium and calcium (PBS, Invitrogen) to remove extracellularly bound NPs. Cells were fixed using 4 % formaldehyde (15 min, BD Biosciences) and rinsed again two times with PBS. Chamber slides with cells and internalized PEGylated AuNPs were wetted with PBS, covered with a coverslip, and sealed with nailpolish to prevent drying.

Cells were imaged at 40× magnification (Plan Fluorite, NA = 0.75, Olympus) using an Olympus BX-41 upright microscope with a CytoViva high-resolution illuminator and a quartz halogen lamp with aluminum reflector (400–1000 nm). The CytoViva Hyperspectral Imaging System was used to collect spectral data across the sample through automated movement of the sample across a transmission diffraction grating spectrograph (Specim) using a X-Y motorized stage (Prior). In order to measure spectra primarily originating from NPs within the cells, darkfield HS images were taken 5 μm above the slide plane (controlled by a Prior motorized z focus drive with 0.002 μm minimum step size; see Additional file 1 of [13] for more details). HS images, which contain full spectral data at each X-Y pixel location, were analyzed using ENVI software (ITT Visual Information Solutions). As we previously reported [13], cell regions of interest (“cell ROIs”) were defined to measure spectra representing the cellular-level optical response. Cell ROIs were defined by tracing around cell boundaries using the polygon ROI tool (see Additional file 1 of [13] for more details). To ensure objectivity in analysis, all cells within a HS image were defined as long as cell boundaries could be unambiguously identified. Spectral data averaged across the pixels of each ROI was extracted by the ENVI software. Spectral data was divided by the normalized lamp spectrum to calibrate for variations in lamp intensity.

### Peak Wavelength Determination

To objectively determine the peak wavelengths of spectra, the peak analyzer function of OriginPro 9.1 Data Analysis and Graphing Software (OriginLab) was employed. Savitzky-Golay smoothing with a window size of 50 was performed, followed by peak finding using a local maximum method with two local points. Spectra that were dominated by cell scattering (characterized by an optical spectrum that monotonically decreases in intensity with longer wavelengths) and had insufficient NP spectral contributions were excluded from analysis since a peak wavelength could not be accurately assessed [13]. Spectra were deemed cell scattering-dominated and not included in analysis if the normalized intensity at 500 nm was 0.95 or higher [13].

### Spectral Broadening Measurement

To calculate the spectral broadness, defined as the width of the spectrum at 95 % of the maximum intensity, a custom MATLAB program was utilized which was discussed in full detail previously [13]. Briefly, spectra were smoothed using a Savitzky-Golay algorithm and shifted downward by a constant value equivalent to 95 % of the spectrum’s maximum intensity. The difference between the two points of zero crossing was then calculated, which represented the spectral width at 95 % of the spectrum’s maximum intensity.

### Characterizing Spectral Shift of PEGylated AuNPs in CPRFM

To measure the shift in NP spectra attributed to protein corona formation or agglomeration in CPRFM prior to cellular uptake, extinction spectra were collected using an Agilent Cary 60 UV-vis spectrophotometer. Spectra were measured for PEGylated AuNPs in water as well as in CPRFM (containing phenol-red free McCoy’s 5A media (HyClone), 1 % penicillin-streptomycin (Sigma-Aldrich) and either 0, 10, 25, or 50 % HuS (Valley Biomedical)) through time. PEGylated 100-nm AuNPs were centrifuged, redispersed in CPRFM, and incubated at 37 °C and 5 % CO_2_ in a cell culture incubator. NPs in water only and CPRFM alone were also kept under same conditions. All spectra were collected with water as the baseline.

### Cellular TEM for Bare and PEGylated AuNPs

Following incubation with NPs, cells were rinsed three times with 1× PBS, fixed with 2.5 % formaldehyde/2.5 % glutaraldehyde in 0.1-M sodium cacodylate buffer (Electron Microscopy Sciences) at room temperature, and kept at 4 °C overnight. After fixation, the samples were washed in 0.1-M cacodylate buffer and treated with 0.1 % Millipore-filtered buffered tannic acid, post-fixed with 1 % buffered osmium tetroxide for 30 min, and stained en bloc with 1 % Millipore-filtered uranyl acetate. The samples were washed several times in water, then dehydrated in increasing concentrations of ethanol, infiltrated, and embedded in Spurr’s low viscosity medium. The samples were polymerized in a 60 °C oven for 2 days. Ultrathin sections were cut in a Leica Ultracut microtome, stained with uranyl acetate and lead citrate in a Leica EM Stainer, and examined in a JEM 1010 transmission electron microscope (JEOL, USA, Inc.) at an accelerating voltage of 80 kV. Digital images were obtained using an AMT Imaging System (Advanced Microscopy Techniques Corp).

### Cellular TEM for PEGylated AuNPs Introduced in Different HuS Conditions

Following incubation with NPs, cells were rinsed three times with 1× PBS and fixed with 2.5 % formaldehyde/2.5 % glutaraldehyde in 0.1-M sodium cacodylate buffer at 4 °C. After fixation, the samples were washed in 0.1-M cacodylate buffer and post-fixed with buffered osmium tetroxide for 30 min. The samples were washed several times in double-distilled water, then dehydrated in increasing concentrations of ethanol, infiltrated, and embedded in Spurr’s low viscosity medium. The samples were polymerized in a 60 °C oven overnight. Ultrathin sections were cut on an RMC MT6000XL microtome and examined unstained on a JEM 1230 transmission electron microscope (JEOL, USA, Inc.) at an accelerating voltage of 80 kV. Digital images were obtained using a US1000 high-resolution digital camera (Gatan, Inc., Pleasanton, CA).

### Statistical Analysis

In comparisons of spectral peak wavelengths among different serum concentration conditions, statistical significance was evaluated by performing one-factor analysis of variance (ANOVA) followed by a post hoc Tukey’s HSD test for multiple comparisons. *P* values less than 0.01 were considered statistically significant. In comparisons made between spectral peak wavelengths (and spectral width) of bare and PEGylated AuNPs following uptake by cells, a two-tailed Student’s *t* test was performed. *P* values less than 0.01 were considered statistically significant.

## Results and Discussion

### PEGylation of Nanoparticles Decreases Spectral Shift Exhibited in Cellular Environment

**Fig. 1:**
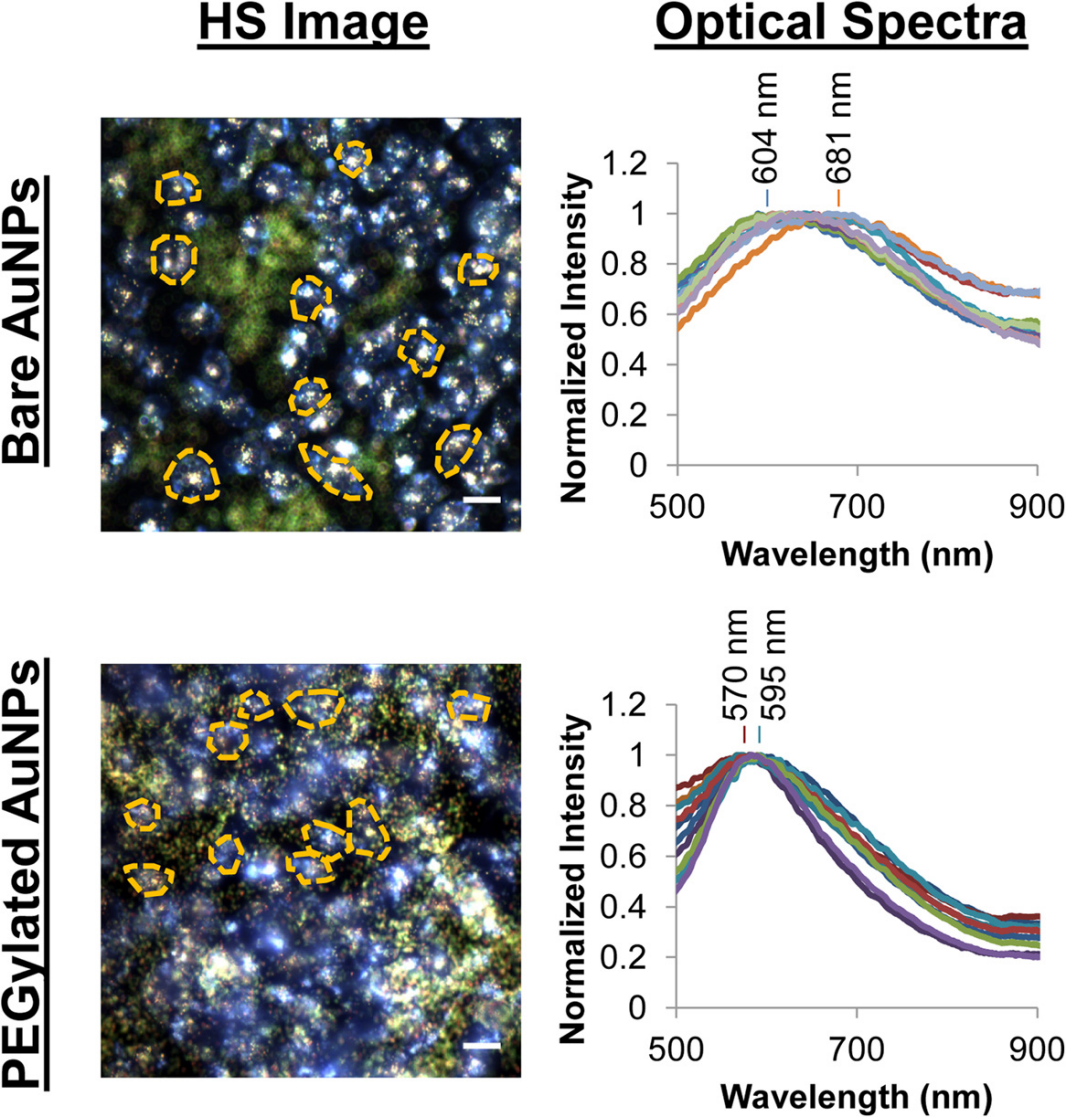
Representative hyperspectral (HS) images and optical spectra showed differences in cellular interaction for bare and PEGylated AuNPs, which corresponded to spectra that differed in peak wavelength and broadness. Shown are representative HS images of Sk-Br-3 cells after exposure to 24 μg/mL of bare or PEGylated AuNPs for 24 h and representative optical spectra extracted from regions of interest (ROIs) defined around individual cells (“cell ROIs”) in the measured HS images (only ten ROIs and only one of the analyzed HS images are shown here for clarity). Scale bar denotes 20 μm. The representative HS image and optical spectra for bare AuNPs are reproduced from ref [13] with permission as a reference for comparison with the representative HS image and optical spectra for PEGylated AuNPs

To study the effect of PEGylation on how the optical properties of nanoparticles change upon cellular uptake, we incubated breast cancer (Sk-Br-3) cells with 100-nm AuNPs functionalized with 5 kDa MW methoxy-terminated poly(ethylene glycol) (mPEG-SH). Cells were incubated with PEGylated AuNPs for 2, 5, 10, or 24 h at 12, 24, 48, or 96 μg/mL exposure doses. Following NP exposure, cells were imaged using darkfield hyperspectral (HS) imaging, in which complete optical scattering spectra can be obtained at each pixel location within the images. We employed our previously reported analysis approach to follow changes in the optical spectra following NP interaction and uptake by the Sk-Br-3 cells [13]. As seen in the representative HS images and corresponding spectra in Fig. 1 for cells exposed either to bare or PEGylated AuNPs for 24 h at a dose of 24 μg/mL, PEGylated NPs and bare NPs interacted with cells in distinctly different ways, with bare NPs forming large clusters in cells. The corresponding spectra (only a subset is shown for clarity) measured from cells exposed to bare and PEGylated NPs differ in both peak wavelength and broadness.

**Fig. 2:**
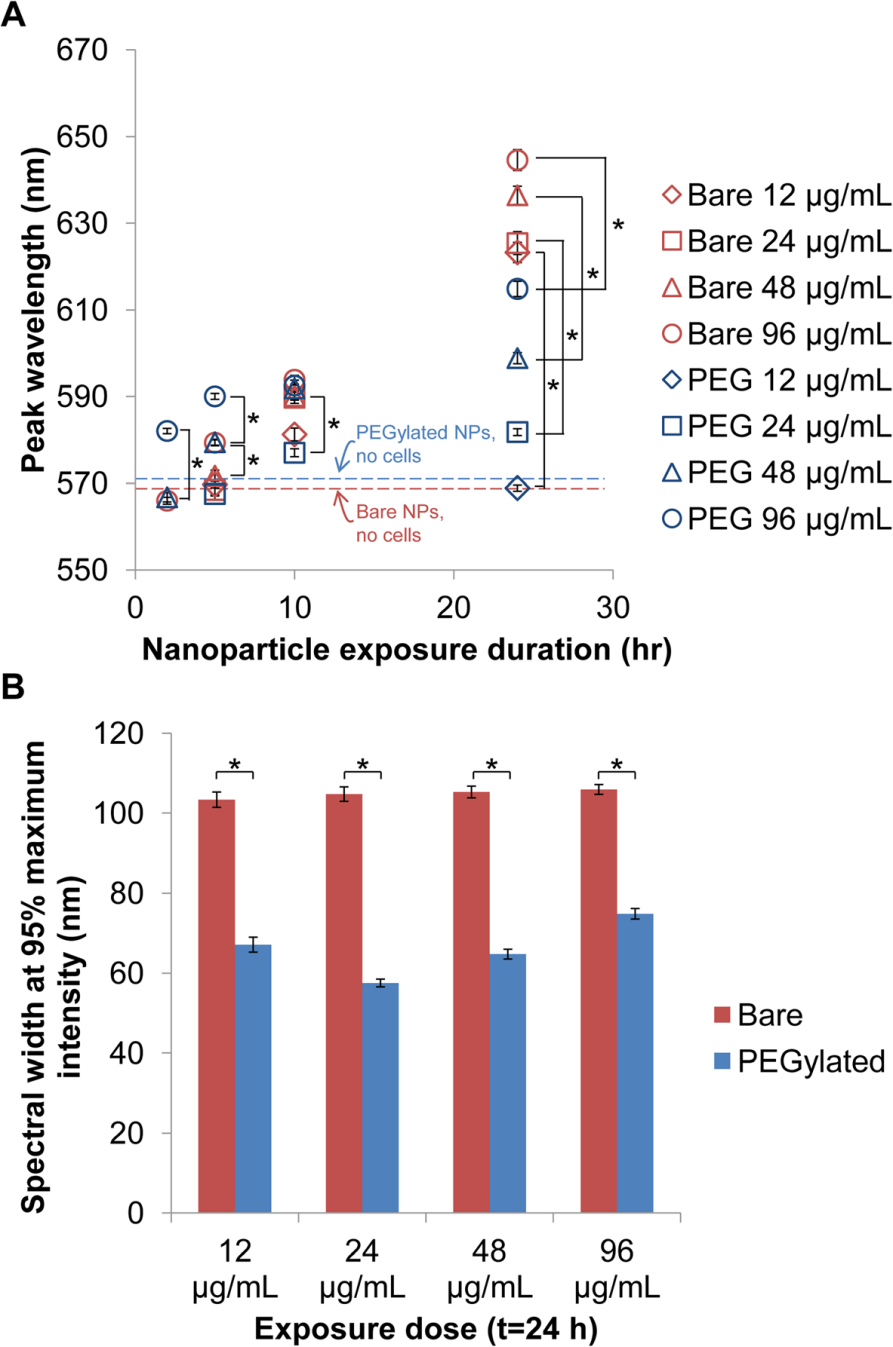
PEGylated AuNPs experienced less spectral shifting and broadening in cells than bare AuNPs, as determined by hyperspectral imaging analysis. **a** Peak wavelengths of cell ROI spectra following exposure of bare and PEGylated AuNPs to Sk-Br-3 cells. *Dotted lines* denote the peak wavelength of UV-vis spectra for bare and PEGylated AuNPs in water in the absence of cells. **b** Spectral width at 95 % maximum intensity after 24 h for bare and PEGylated AuNPs. *Error bars* indicate SEM. Spectral peak wavelengths were measured from more than 35 cell ROIs for each condition. * indicates statistically significant difference (*p* < 0.01) based on two-tailed Student’s *t* test. Data for bare AuNPs are re-plotted here from ref [13] with permission as a reference for comparison with data for PEGylated AuNPs

Quantitative analysis resulting from extracting the spectra from more than 35 individual cells in each condition determined that NPs exhibit less spectral shift following cellular uptake when they are functionalized with PEG as compared with when they are bare (Fig. 2a). While spectra for bare AuNPs shifted by as much as 79 ± 24 nm between 2 and 24 h of incubation, spectra for PEGylated AuNPs shifted a maximum of 33 ± 12 nm (Fig. 2a). The spectral shift measured for PEGylated AuNPs was greater than can be attributed to changes in local surrounding dielectric constant associated with the formation of a protein corona on the PEGylated AuNP surface in cell culture media prior to cell uptake (See Additional file 1: Figure S1) or the contribution of the cellular refractive index of 1.33~1.38 [13, 36, 37]. As seen in Fig. 2a, 12 to 96 μg/mL exposure doses of bare AuNPs resulted in spectra with peak wavelengths ranging from 623 ± 25 to 645 ± 23 nm after 24-h incubation with cells, while the same exposure doses of PEGylated AuNPs only resulted in spectra with peak wavelengths ranging from 569 ± 4 nm to 615 ± 12 nm.

The smaller spectral shift experienced by PEG-coated AuNPs compared with bare AuNPs may be attributed to the differences in cellular interaction and intracellular trafficking of the two types of AuNPs. PEGylated AuNPs interface differently with cells than do bare AuNPs due to the sterically hindering polymer chains presented on their surface and resulting different surface charge [38, 39]. The PEG layer has been found not only to reduce serum protein adsorption but also to impact the composition of the NP’s protein corona [22], which influences where NPs are transported within cells. Compared with bare AuNPs, PEGylated AuNPs have been shown to experience decreased cellular uptake and distinct patterns of intracellular distribution and agglomeration [38, 39].

**Fig. 3:**
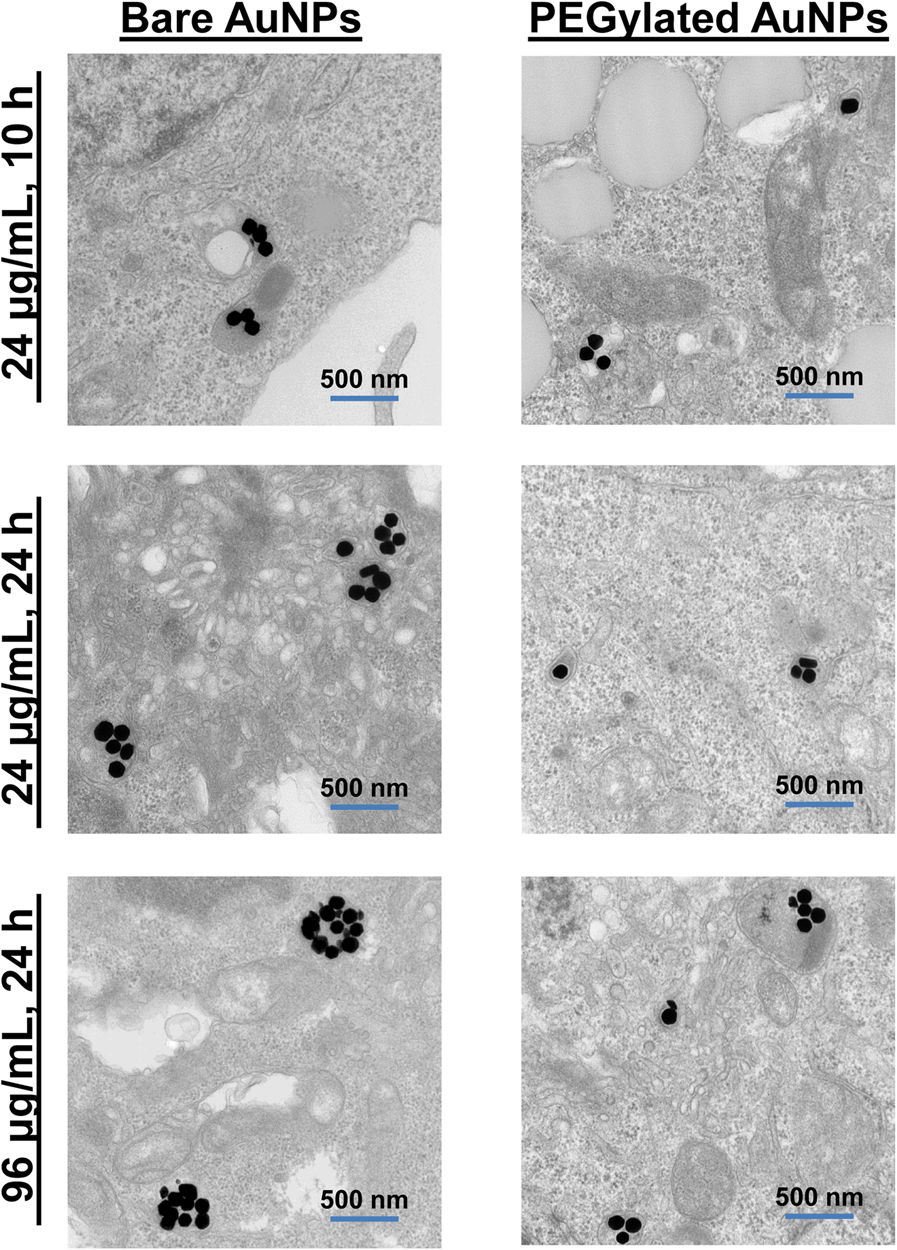
Representative cellular TEM images showed differences in cellular uptake and agglomeration for bare and PEGylated AuNPs. PEGylated NPs associated less with cells, resulting in fewer NPs per NP cluster (NPC) within cells. The smaller NPCs formed by PEGylated AuNPs support the smaller shift and broadening of PEGylated AuNP spectra in a cellular environment that was measured by HS imaging

Indeed, cellular TEM imaging showed that the decreased shifts for PEGylated AuNPs in cells correlated with the formation of smaller intracellular NP clusters (NPCs) as compared with bare AuNPs under the same exposure dose and time conditions (Fig. 3). For example, following exposure of cells to 24 μg/mL of bare or PEGylated AuNPs for 24 h, vesicles showed 1–5 NPs in each cell slice for bare NPs as compared with 1–3 NPs for PEGylated AuNPs (Note: TEM images are taken from cell slices, and thus, the actual number of NPs per vesicle is larger due to the 3D nature of vesicles within cells. The number of NPs within vesicles from cell slices is provided for relative comparison. Cell slices were consistently taken approximately 1.5 μm into the cell). Likewise, at the 96 μg/mL exposure dose, larger NP clusters (NPCs) were observed in cells exposed to bare AuNPs than in cells exposed to PEGylated AuNPs (~8–12 NPs per NPC vs. 2–6 NPs per NPC in TEM cell slices; see Fig. 3, Additional file 1: Figure S2, and Additional file 1 of [13]).

Our observations are consistent with previous studies in human alveolar epithelial cells and mouse macrophage cells which reported that PEGylated NPs formed smaller intracellular agglomerates than did bare AuNPs [22, 39] and experienced decreased cellular uptake [40]. However, in comparison to a previous study that observed that PEGylated NPs were better dispersed and spaced out from one another within vesicles of macrophage cells [22], we did not observe a significant difference in distance among particles for the PEGylated NPs compared with for the bare AuNPs within endosomes in the Sk-Br-3 cells used in our study (Fig. 3). The decreased spectral shift for PEGylated AuNPs that we observed appears to be mainly due to the smaller size of AuNP clusters that form when PEGylated AuNPs are uptaken by cells rather than increased inter-particle distance (and thus a decreased coupling of nanoparticle plasmon resonances [23]) provided by the PEG layer.

### PEGylated AuNPs Experience Less Spectral Broadening Following Cellular Uptake than Bare AuNPs

The broadness of spectra determines the range of wavelengths at which a certain intensity of optical response (absorption or scattering of light) will be achieved. Biomedical applications that depend on multiplexing of NPs resonant at different wavelengths must utilize NPs with narrow spectral widths in order to prevent unintentional excitation of other NP populations when illuminating with a particular wavelength [3, 13, 41]. Therefore, we compared the broadening of spectra after the introduction of bare and PEGylated AuNPs to cells. Since the spectra were asymmetric, with the left side of the peak often not dropping below 50 % of the maximum intensity, broadness could not be defined as the full width half maximum (“FWHM”) [13, 42]. We defined broadness by measuring the spectral width at 95 % of the maximum intensity (“spectral width”) [13]. As seen in Fig. 2b, PEGylated AuNP spectra were significantly narrower than bare AuNP spectra following cellular interaction. Whereas the spectral width of bare AuNPs ranged from 103.4 ± 20.3 nm to 105.9 ± 11.7 nm for all exposure doses after 24 h of cell incubation, the spectral width for PEGylated AuNPs ranged from 57.5 ± 7.4 nm to 74.9 ± 8.9 nm after 24 h incubation.

The decreased broadening for PEGylated AuNPs is likely because of the decreased heterogeneity in NPC sizes within cells. Averaging of the various spectra from intracellular NPCs of different sizes and peak wavelengths will result in a broader spectrum for cells that contain NPCs of diverse sizes [13]. TEM images suggested that for PEGylated AuNPs, most intracellular vesicles contained only 1 to 3 NPs up to a maximum of 8 NPs per vesicle (within a cell slice) (Fig. 3, Additional file 1: Figure S2). In comparison, a greater range of NPC sizes (1 to 29 NPs per vesicle) was observed for bare AuNPs (Fig. 3, Additional file 1 of [13]). This difference in the range of NPC sizes found within cells incubated with PEGylated or bare AuNPs is again likely to stem from the decreased uptake and unique cellular uptake/intracellular trafficking routes taken by PEGylated AuNPs (as shown through inhibition studies previously) [38, 39].

In summary, these results show that PEGylated AuNPs experience smaller spectral shifting and decreased broadening in a cellular environment compared with bare AuNPs, likely due to lower cellular uptake and formation of smaller intracellular clusters. Such results suggest that PEGylating NPs can be utilized as a method for mitigating changes to NPs’ spectral profiles; however, we also find that PEGylation does not completely prevent spectral shifting and broadening, and that the extent of spectral shifting and broadening is time- and exposure dose-dependent (Fig. 2).

### Serum Concentration Impacts Shift Magnitude of NP Spectra in Cells

**Fig. 4:**
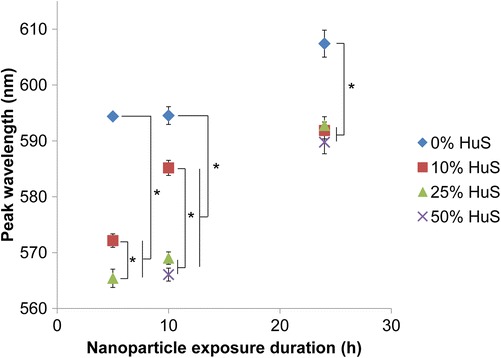
Serum concentration impacted the magnitude of spectral shift experienced by PEGylated AuNPs in cellular environment. Peak wavelengths of spectra measured from HS imaging are displayed for Sk-Br-3 cells incubated with PEGylated AuNPs in 0, 10, 25, or 50 % HuS conditions for 5, 10, or 24 h. *Error bars* indicate SEM for spectral peak wavelengths measured from at least 50 cell ROIs for each condition. PEGylated AuNPs exposed to cells in serum-free conditions exhibited spectra with peak wavelengths that were significantly greater than spectra for NPs exposed to cells in serum-containing conditions (10, 25, or 50 % HuS), suggesting a greater spectral shift. Comparing spectral shifts exhibited by NPs exposed to cells in different serum-containing conditions (10, 25, and 50 % HuS), shifts were initially smaller at higher HuS conditions (25 and 50 % HuS) as compared with at the 10 % HuS concentration typically used in in vitro studies. However, after 24 h of exposure, shifts became comparable and were not significantly different among the serum-containing conditions. * indicates statistically significant difference (*p* < 0.01) based on ANOVA followed by Tukey’s post hoc HSD test. Note: No peak wavelength could be calculated at *t* = 5 h for NPs exposed to cells at 50 % HuS since there was insufficient NP signal and cell scattering dominated the spectrum (for more information, see [13]). *Error bar* for 0 % HuS at *t* = 5 h is smaller than the blue diamond symbol and therefore not visible

Since plasmonic NPs are exposed to different biological environments depending on their application (e.g., low serum concentration for in vitro applications, but high-serum concentration for in vivo applications), we further studied the effect of serum concentration on the spectral changes experienced by plasmonic NPs in cells. We chose 0 and 10 % HuS concentrations since plasmonic NPs are used under serum-free and serum-containing conditions in vitro. We chose 25 and 50 % HuS to represent higher serum conditions closer to those found in vivo. Consistent with a previous study, we did not test concentrations higher than 50 % HuS so that we could continue providing nutrients and buffering agents from the base cell culture media to the cells [35]. Prior to introduction of NPs, cells were slowly weaned to the 10, 25, or 50 % serum conditions through the course of multiple passages in order to ensure that measured effects were not due to cellular shock from a new environment. Cells cultured under these serum conditions were exposed to NPs suspended in media containing the respective serum concentrations and visualized through HS imaging. Quantitative analysis of HS data showed that at early times (5 and 10 h), serum concentration impacted the magnitude of spectral shift experienced by AuNPs in cells; however, after 24 h, cellular uptake-associated shifting in the NP spectra was comparable among the 10, 25, and 50 % HuS conditions (Fig. 4).

At 5 and 10 h, there were distinct differences in the peak wavelengths of spectra for NPs introduced to cells cultured in 0, 10, 25, and 50 % HuS conditions. NPs introduced to cells in serum-free (0 % HuS) conditions exhibited spectra with significantly longer peak wavelengths than did NPs exposed to cells in serum-containing (10, 25, and 50 % HuS) conditions. Serum concentration has recently been shown to greatly impact the composition and thickness of the protein corona formed on the NP surface as well as the cellular uptake of NPs [29, 31, 35]. For instance, Payne et al. showed that incubation of NPs with serum proteins could result in either increased or decreased cellular binding of cationic or anionic NPs as a result of the protein corona formed on the NP surface [43]. The protein corona of anionic NPs contained serum albumin, causing the NPs to bind to cells primarily through the albumin receptor and resulting in decreased cell binding as they competed with free albumin proteins to bind the albumin receptor. In comparison, the protein corona of cationic NPs contained a denatured form of serum albumin, which caused NPs to interact with cells through scavenger receptors and experience increased uptake [43].

Here in our study, our PEGylated AuNPs were slightly anionic (see Additional file 1: Table S1), and they experienced smaller spectral shifts in serum-containing media (10, 25, and 50 % HuS) compared with serum-free media (0 % HuS) (Fig. 4). These results agree with Payne’s study in that the presence of serum for our anionic AuNPs likely resulted in the formation of a specific protein corona that caused the NPs to engage particular cell receptors and take a distinct route of internalization and intracellular transport with less cellular uptake. Decreased cellular uptake of the NPs can result in each intracellular vesicle containing fewer NPs, leading to a smaller chance for plasmonic coupling and spectral shift.

**Fig. 5:**
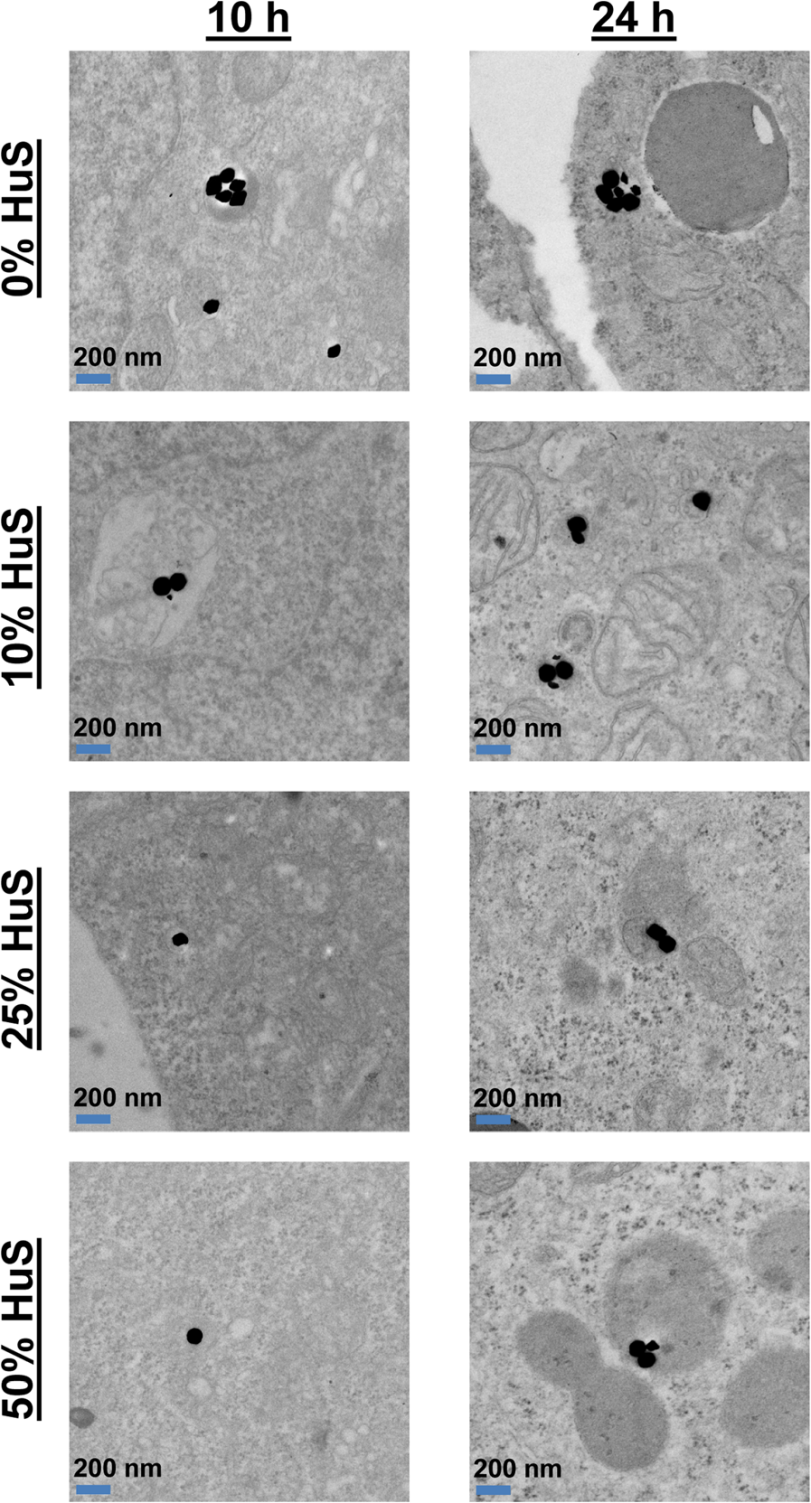
Representative cellular TEM images showed the relative number of PEGylated AuNPs within intracellular vesicles 10 and 24 h after incubation with Sk-Br-3 cells in various serum concentration conditions. At 10 h, the cells exposed to PEGylated AuNPs in serum-free conditions were observed to have the highest number of AuNPs per vesicle, followed by decreasing numbers of AuNPs per vesicle as the serum concentration increased. After 24 h, the largest numbers of AuNPs were found in vesicles when AuNPs were introduced to cells in serum-free conditions, while a comparable number of AuNPs was found in vesicles in the serum-containing conditions (10, 25, 50 % HuS)

At 5 and 10 h of NP exposure, AuNPs also showed decreased spectral red-shifting as serum concentration increased from 10 to 25 and 50 % HuS (Fig. 4). Previous studies have demonstrated that increased serum concentrations can lead to decreased cellular uptake of functionalized AuNPs or amine-modified polystyrene NPs [29, 35]. Therefore, decreased cellular uptake of NPs by cells incubated in higher serum concentrations may be one factor explaining the minimal change in spectral peak wavelength for NPs incubated with cells in the high-serum (25 and 50 % HuS) conditions at 5 and 10 h. In addition to cellular uptake, the critical factor determining the extent of shift in the NP spectra is the configuration of NP agglomerates in intracellular vesicles. In Fig. 5, representative TEM images of cells at *t* = 10 h show that at the highest serum concentrations (25 and 50 % HuS), fewer NPs (1-2 NPs) were found within each intracellular vesicle as compared with for cells exposed to NPs in 10 % HuS or serum-free (0 % HuS) conditions (see also Additional file 1: Figure S3). The decreased number of NPs found within intracellular vesicles for cells cultured in higher serum concentration conditions is consistent with the smaller shift in the NP spectra that was measured (Fig. 4).

Interestingly, after 24 h of incubation of the PEGylated AuNPs with cells, there was no longer a significant difference in the spectral peak wavelength among the 10, 25, and 50 % HuS conditions (Fig. 4). We hypothesize that this is due to “cellular conditioning” of the extracellular media through secretion of solutes, proteins, and ions by cells. Albanese and Chan recently showed that this natural ongoing secretion of proteins and molecules by cells into the extracellular media (for communication with other cells) causes a change in NPs’ protein corona and aggregation of NPs outside of cells [44–46]. Albanese and colleagues showed that between 8 and 24 h of cellular incubation, the composition of the NP’s protein corona begins to include more cell-secreted proteins and less serum proteins from the original media, resulting in changes in NP aggregation and cellular uptake [44]. This supports our finding that serum concentration impacted the magnitude of spectral shift experienced by AuNPs at early time points of incubation with cells but did not engender differences in spectral shift after 24 h of incubation. It is possible that by 24 h of incubation, cell-secreted proteins and biomolecules have displaced a portion of the original protein corona and made the protein corona of the NPs in 10, 25, and 50 % HuS conditions, more comparable in composition. A second possible reason or contributing factor for the disappearance of significant differences in the spectral peak wavelength among the different HuS concentration conditions after 24 h is that the protein corona evolved to a common composition over time for NPs incubated in the different serum conditions. Monopoli et al. showed that such an evolution in the protein corona over 24 h to a more uniform composition no longer dependent on the plasma concentration occurred for sulfonated polystyrene NPs [31]. A third explanation is that NPs exposed to 10, 25, or 50 % HuS conditions could undergo different paths of cellular uptake and intracellular transport due to their distinct protein coronas, explaining the earlier differences in spectral shift and slight differences in NPC size seen in TEM images; however, after 24 h, NPs in high-serum concentration conditions may have had enough time to “catch up” in cellular internalization to reach NPC sizes similar to those of NPs incubated with lower concentrations of serum. Indeed, TEM images of cells incubated with NPs in 10, 25, and 50 % HuS showed that similar NPC sizes resulted after 24 h, which can be due to a combination of these three reasons.

Together, our results suggest that for PEGylated AuNPs, the serum concentration has a greater effect on the extent of spectral shift experienced by PEGylated AuNPs at early times following introduction to cells, but this effect diminishes with longer incubation time. However, significant larger spectral shifting is achieved for NPs in serum-free conditions compared with serum-containing conditions. As a result, we find that the biological environmental conditions of the applications in which plasmonic nanoparticles are used—whether a serum-free or serum-containing environment—will impact the extent of the change in their optical spectra upon cellular interaction, likely as a result of the formation of a different protein corona and engaging different cellular receptors for uptake and intracellular trafficking.

## Conclusions

In this study, we examined how design parameters, such as PEGylation of NPs and serum concentrations used, impact the extent that the spectra of plasmonic NPs change when plasmonic NPs are introduced into a cellular environment. We found that PEGylation decreases the magnitude of spectral shift and spectral broadening. Changes in the spectra of plasmonic AuNPs in cells is also dependent on the serum concentration in which the NPs are introduced to the cells. In serum-free conditions, NP spectra shift significantly more, and when NPs are exposed to cells for short periods of time, spectral shifts are suppressed in higher serum concentration environments. Finally, after 24 h of NP-cell exposure, it appears that the serum concentration of the extracellular media does not have a significant effect on the extent of spectral shifting either due to evolution of the protein corona to a common composition or due to NPs reaching a similar plateau in intracellular agglomeration after 24 h despite initial differences in cellular uptake and intracellular transport.

These findings suggest that the spectral shifting of plasmonic NPs in biological environments can be engineered when designing nanoparticles for biomedical applications. PEGylation of NPs can decrease spectral shifting and maintain a narrow spectral width, which may be important for applications demanding narrow spectral outputs to minimize spectral overlap of multiplexed NPs. Furthermore, spectral shifts are less pronounced when PEGylated AuNPs are employed in high-serum environments. While these trends may differ depending on particle type and cell type, our findings offer an initial framework for considering the effect of design parameters on the final spectral shifting in cells when designing plasmonic nanoparticles for medical applications. Furthermore, we have demonstrated the use of darkfield HS imaging for identifying how NP design parameters and environmental factors impact the spectral shift plasmonic NPs experience in cellular environments. Continued work towards elucidating the cellular interaction, cellular uptake, intracellular trafficking, and intracellular agglomeration of AuNPs in parallel with further characterization of how other design parameters impact plasmonic NP spectral changes in biological environments will enable the precise design of plasmonic NPs capable of achieving a targeted optical response and fate in biomedical systems.

## Abbreviations

AuNPs, gold nanoparticles; CPRFM, complete phenol-red free media; HS, hyperspectral; HuS, human serum; NPs, nanoparticles; NPC, NP cluster; PEG, poly(ethylene) glycol; ROI, region of interest; TEM, transmission electron microscopy

## Additional file

Additional file 1: Supplementary Data. A document containing four supplementary figures and one supplementary table. UV-vis absorbance readings; zeta potential measurements of bare and PEGylated AuNPs; and additional cellular TEM images. (PDF 4592 kb)
