# Supplementary material for: Changes in Optical Properties of Plasmonic Nanoparticles in Cellular Environments are Modulated by Nanoparticle PEGylation and Serum Conditions
Source: Nanoscale Res Lett. 2016 Jun 18;11:303. doi: 10.1186/s11671-016-1524-4 (PMC4912538; doi:10.1186/s11671-016-1524-4)
Supplement: Additional file 1: — Supplementary Data. A document containing four supplementary figures and one supplementary table. UV-vis absorbance readings; zeta potential measurements of bare and PEGylated AuNPs; and additional cellular TEM images. (PDF 4592 kb) [file 11671_2016_1524_MOESM1_ESM.pdf]

Additional File 1: Supplementary Data

Changes in Optical Properties of Plasmonic Nanoparticles in Cellular Environments are Modulated by Nanoparticle PEGylation and Serum Conditions

Allen L. Chen,[1] *Meredith A. Jackson,*[1] *Adam Y. Lin,*[1] *Elizabeth R. Figueroa,*[1] *Ying S. Hu,*[2] *Emily Reiser,*[1] *Vishwaratn Asthana,*[1] *Joseph K. Young,*[3] *Rebekah A. Drezek*[1,3,]*

[1]Department of Bioengineering, Rice University, Houston, TX 77005

[2]Waitt Advanced Biophotonics Center, Salk Institute for Biological Studies, La Jolla, CA 92037

[3]Department of Electrical and Computer Engineering, Rice University, Houston, TX 77005

Contents

1. Characterizing PEGylated AuNP spectral shift in media before cell internalization 2
2. Additional cellular TEM images: varying exposure dose & time 3
3. Zeta potential measurement of bare and PEGylated 100 nm AuNPs 6
4. Additional cellular TEM images: varying serum concentration at t=10 h 7
5. Additional cellular TEM images: varying serum concentration at t=24 h 11

1. Characterizing PEGylated AuNP spectral shift in media before cell internalization

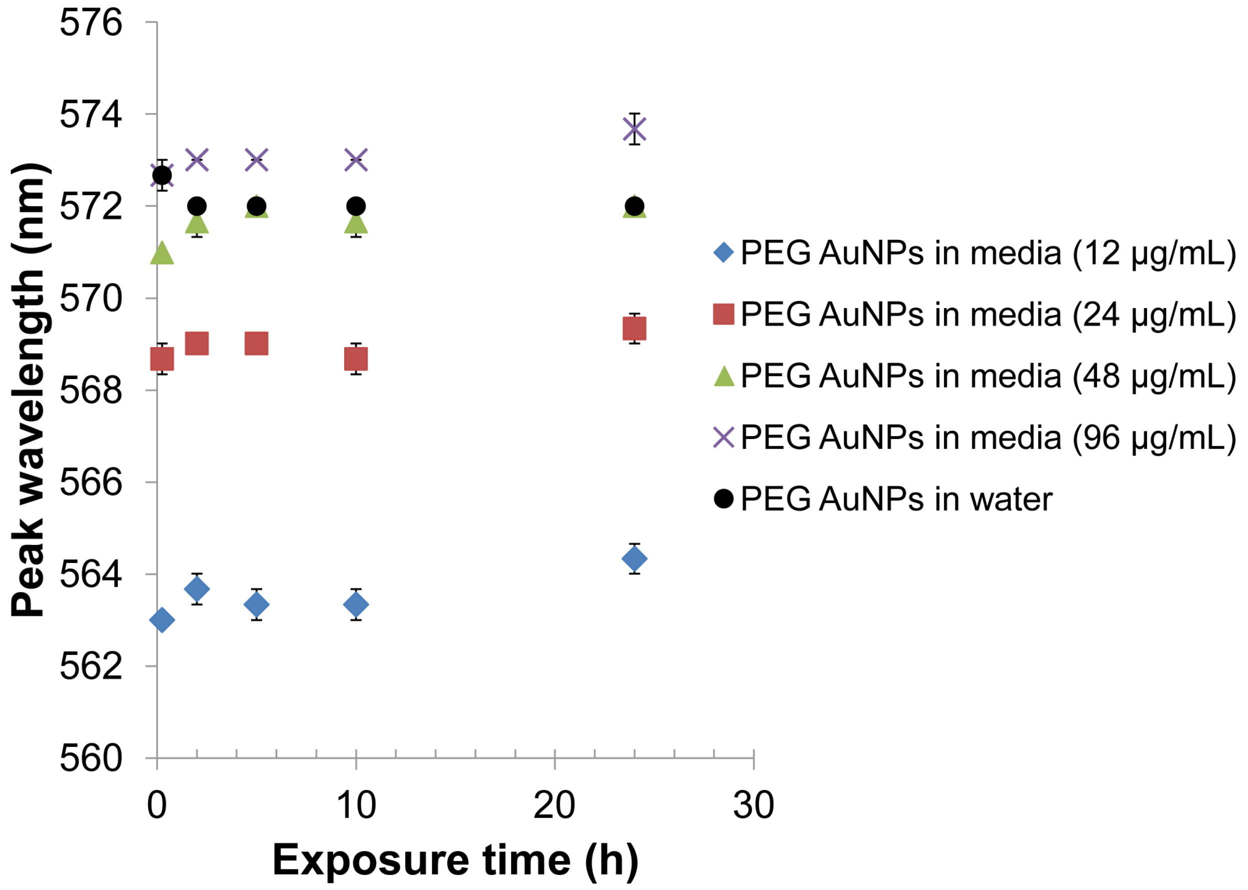

Figure S1. UV-vis absorbance readings show no more than 1 nm in shift of spectra after incubation in 10% HuS cPRFM over 24 h. PEGylated NPs in water serve as a comparative reference. At each exposure dose (12, 24, 48, 96 μg/mL), PEGylated AuNPs in 10% HuS CPRFM did not exhibit a peak wavelength shift of more than 1 nm. The peak wavelengths differ among PEGylated AuNPs at different exposure doses because of the influence of cPRFM components on the spectra. At high doses of PEGylated AuNPs, the contribution of the cPRFM on the spectra are minor. At lower doses of PEGylated AuNP, the AuNP spectral contribution is lower, and the cPRFM spectral contribution causes the overall spectral peak to be slightly blue-shifted (shifted to shorter wavelengths). Nonetheless, within each exposure dose, there is no more than 1 nm of culture media-related shift in the spectra of PEGylated AuNPs prior to cellular uptake, which is insufficient to account for the shifted spectra measured in cells after cellular uptake by HS imaging.

2. Additional cellular TEM images: *varying exposure dose & time*

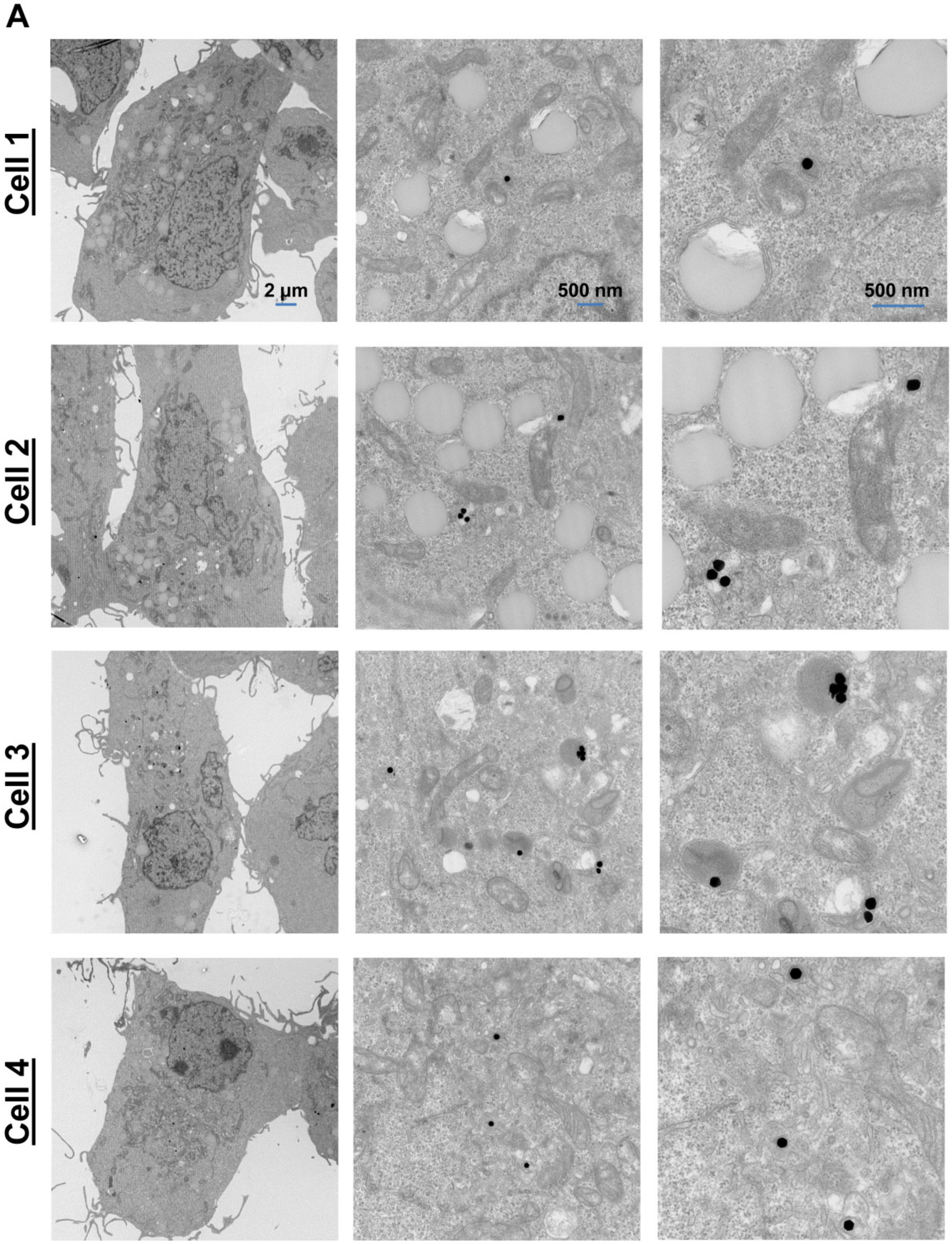

Figure S2A. Additional TEM images of Sk-Br-3 cells following 10 h incubation with 24 µg/mL 100 nm PEGylated AuNPs. TEM images of multiple cells are shown to provide representative sampling of the relative distribution and number of NPs in each cluster and number of clusters per cell (in the two-dimensional slice).

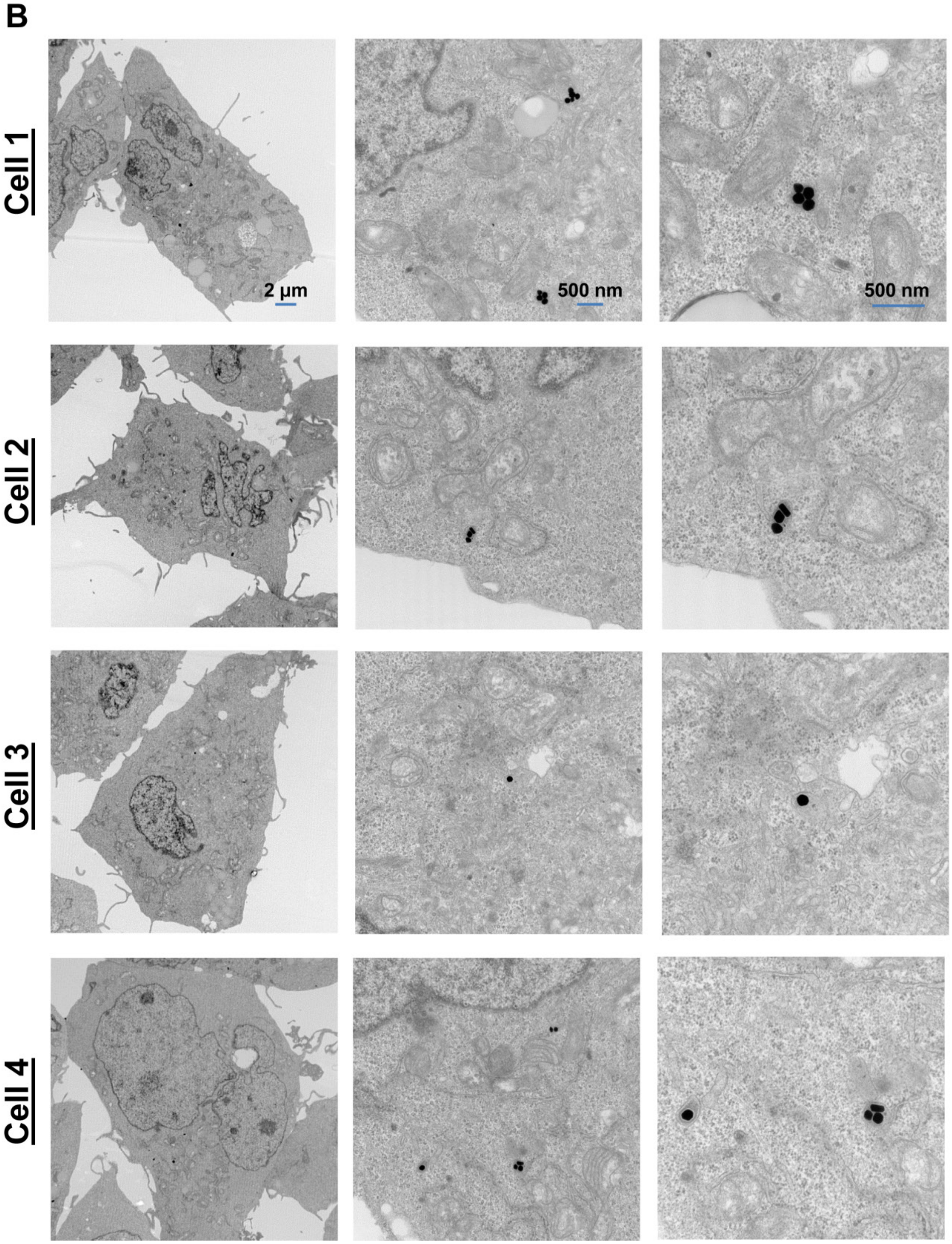

Figure S2B. Additional TEM images of Sk-Br-3 cells following 24 h incubation with 24 µg/mL 100 nm PEGylated AuNPs. TEM images of multiple cells are shown to provide representative sampling of the relative distribution and number of NPs in each cluster and number of clusters per cell (in the two-dimensional slice).

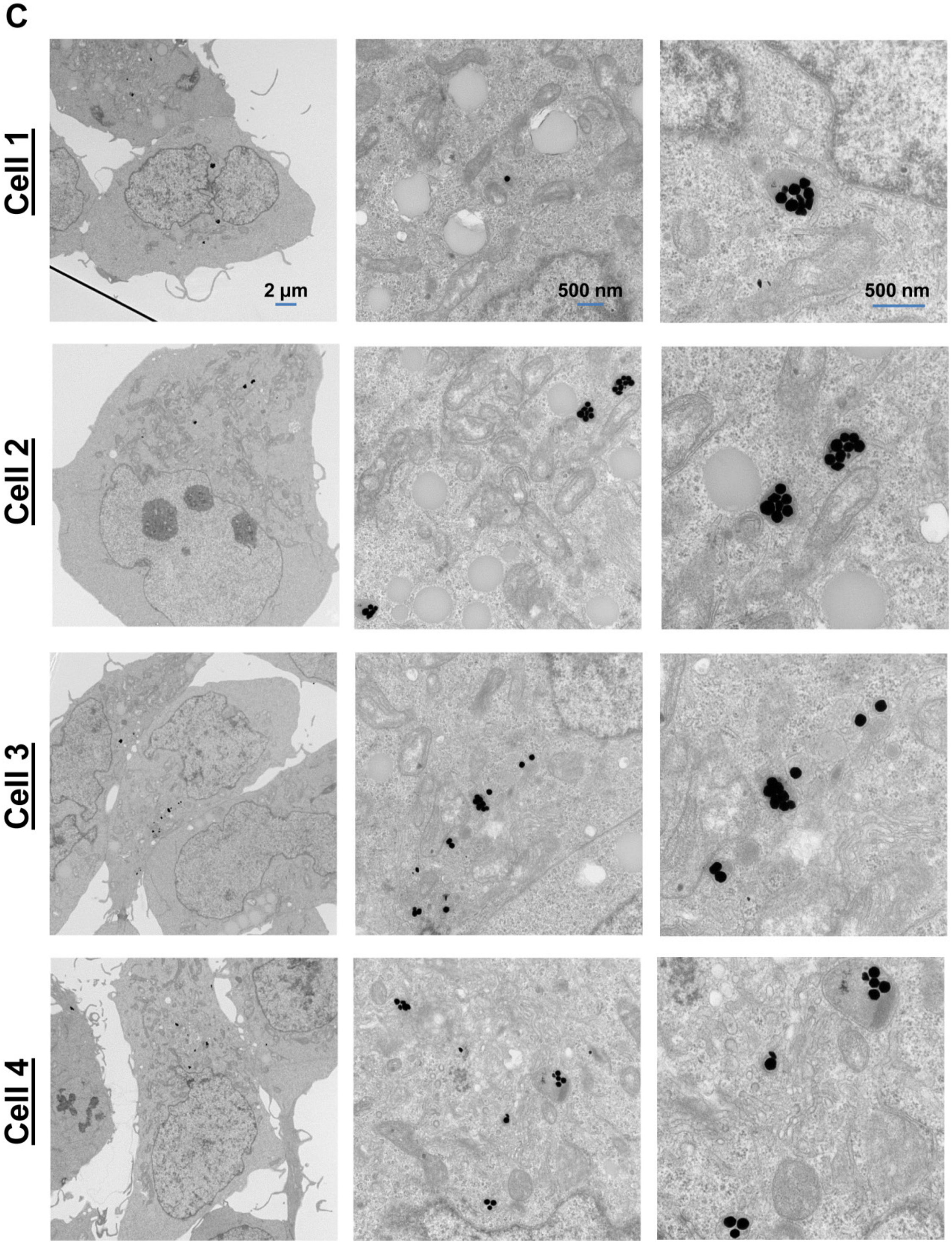

Figure S2C. Additional TEM images of Sk-Br-3 cells following 24 h incubation with 96 µg/mL 100 nm PEGylated AuNPs. TEM images of multiple cells are shown to provide representative sampling of the relative distribution and number of NPs in each cluster and number of clusters per cell (in the two-dimensional slice).

3. Zeta potential measurement of bare and PEGylated 100 nm AuNPs

The zeta potential for bare and PEGylated 100 nm AuNPs was measured using a Malvern Zetasizer Nano ZS at 25°C. Values are reported as mean ± standard deviation.

Table S1. Zeta potential for bare and PEGylated 100 nm AuNPs measured in water.

	Hydrodynamic Diameter (nm)	Zeta Potential (mV)
**Bare AuNPs**	107.0 ± 0.6	-45.4 ± 0.4
**PEGylated AuNPs**	123.3 ± 0.03	-7.2 ± 0.7

4. Additional cellular TEM images: *varying serum concentration at t=10 h*

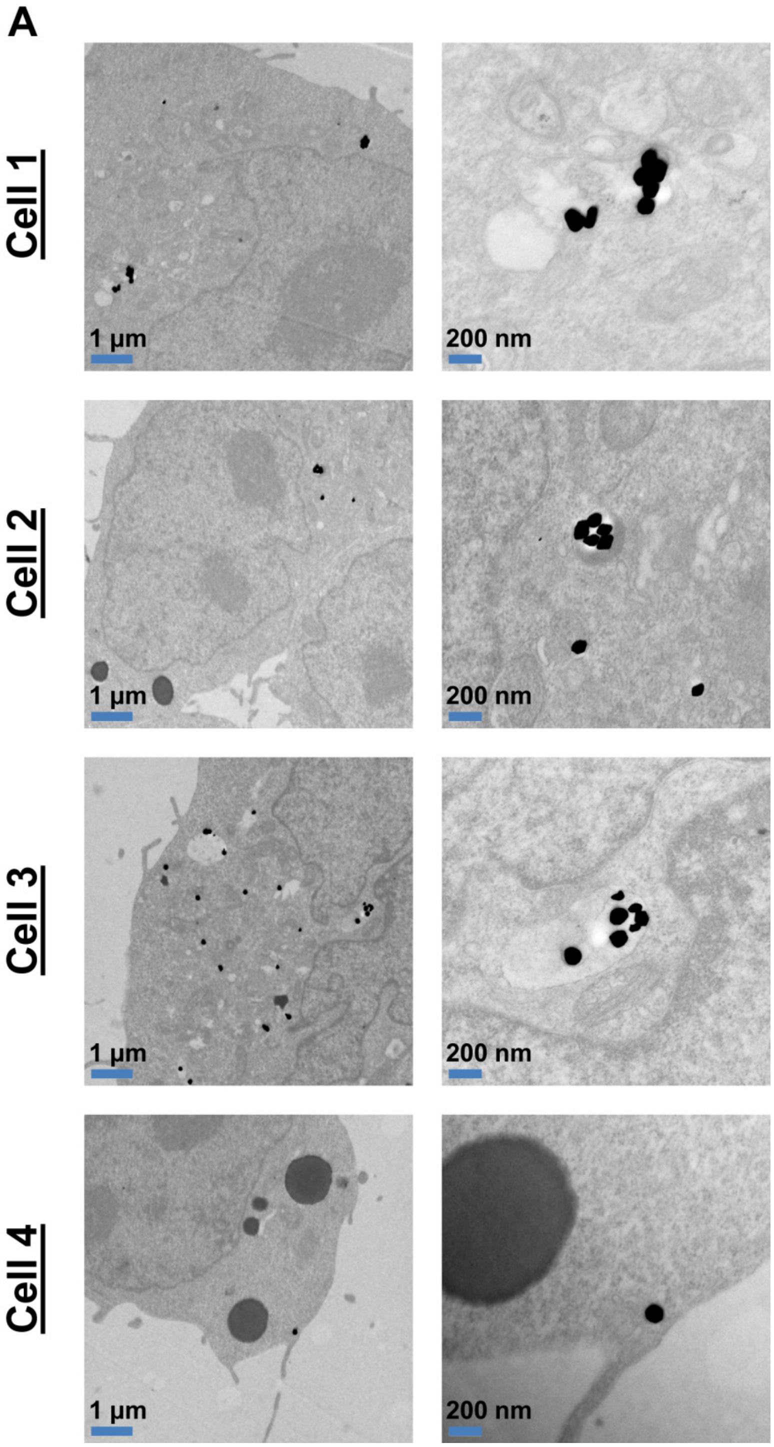

Figure S3 Additional TEM images of Sk-Br-3 cells following 10 h incubation with 24 μg/mL 100 nm PEGylated AuNPs in 0% HuS. TEM images of multiple cells are shown to provide representative sampling of the relative distribution and number of NPs in each cluster (in the two-dimensional slice).

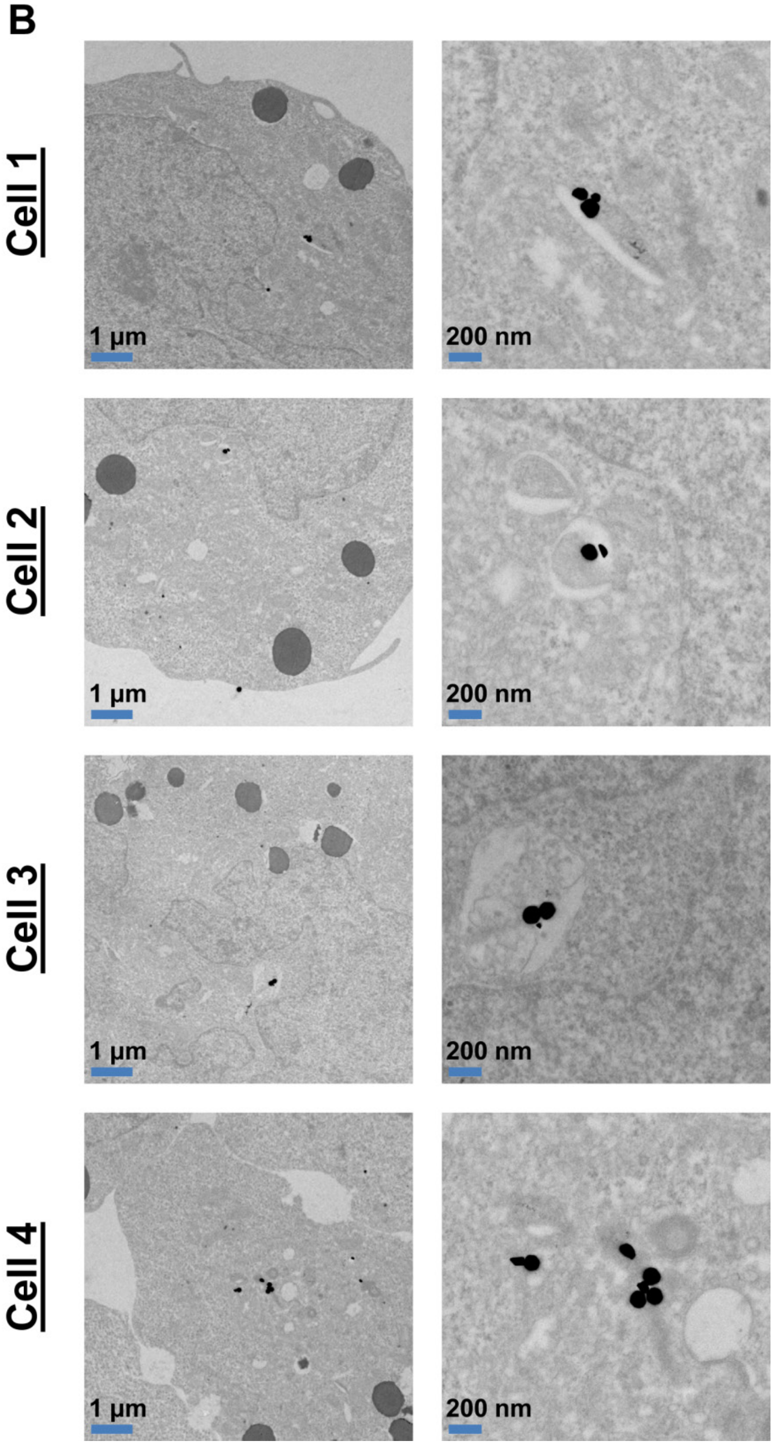

Figure S3 Additional TEM images of Sk-Br-3 cells following 10 h incubation with 24 μg/mL 100 nm PEGylated AuNPs in 10% HuS. TEM images of multiple cells are shown to provide representative sampling of the relative distribution and number of NPs in each cluster (in the two-dimensional slice).

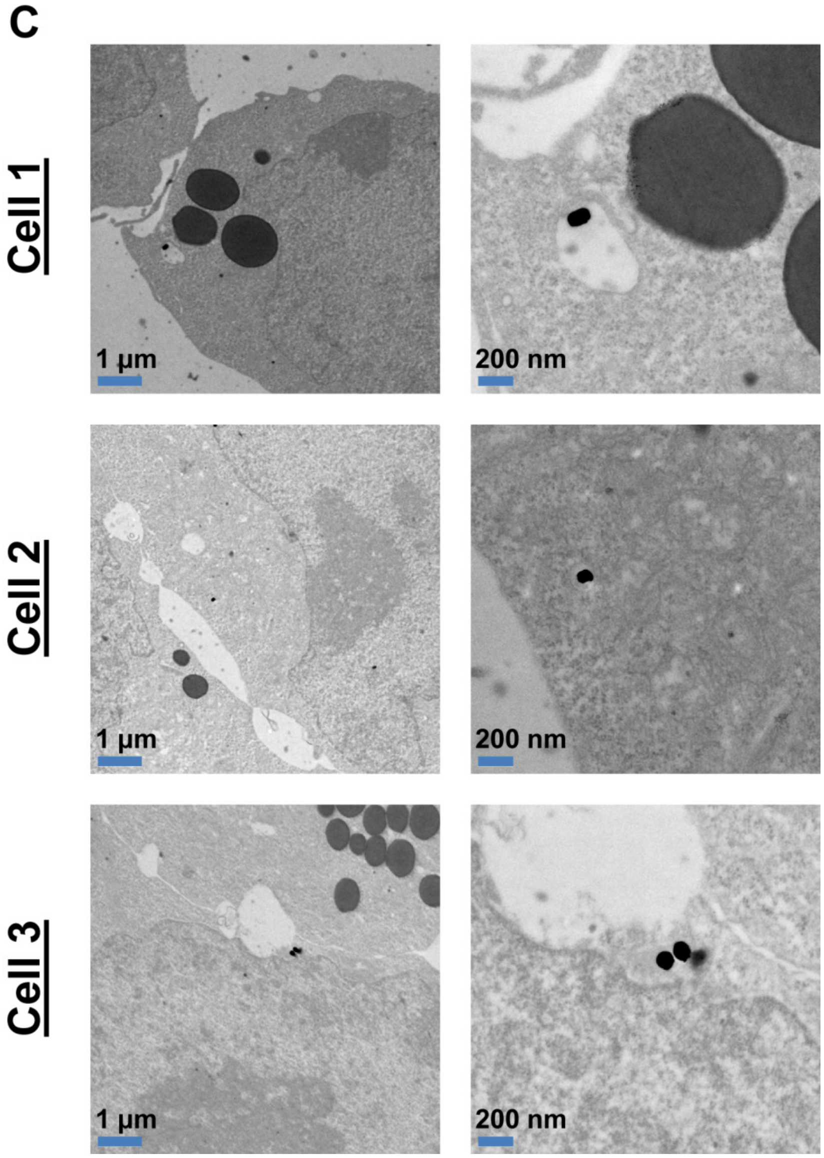

Figure S3 Additional TEM images of Sk-Br-3 cells following 10 h incubation with 24 µg/mL 100 nm PEGylated AuNPs in 25% HuS. TEM images of multiple cells are shown to provide representative sampling of the relative distribution and number of NPs in each cluster (in the two-dimensional slice).

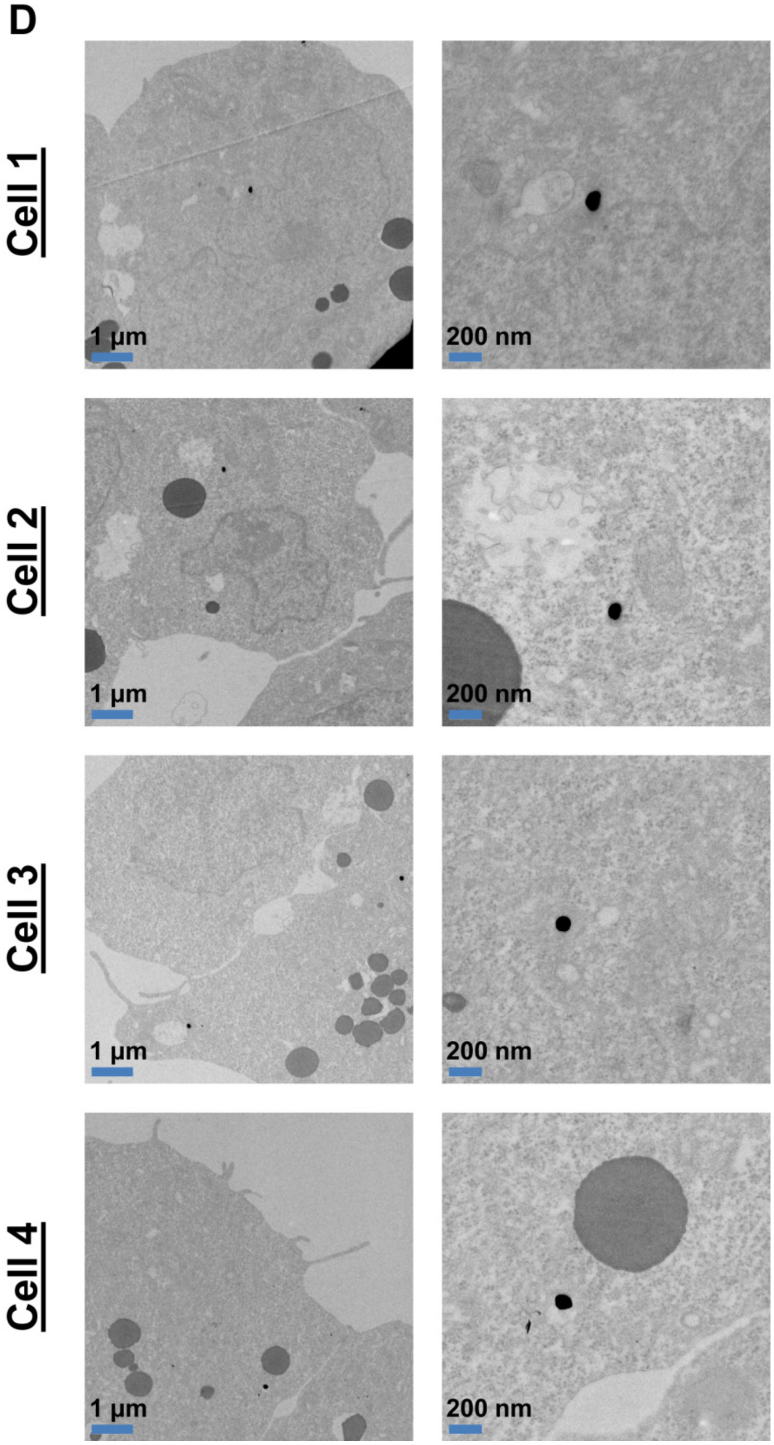

Figure S3 Additional TEM images of Sk-Br-3 cells following 10 h incubation with 24 µg/mL 100 nm PEGylated AuNPs in 50% HuS. TEM images of multiple cells are shown to provide representative sampling of the relative distribution and number of NPs in each cluster (in the two-dimensional slice).

5. Additional cellular TEM images: *varying serum concentration at t=24 h*

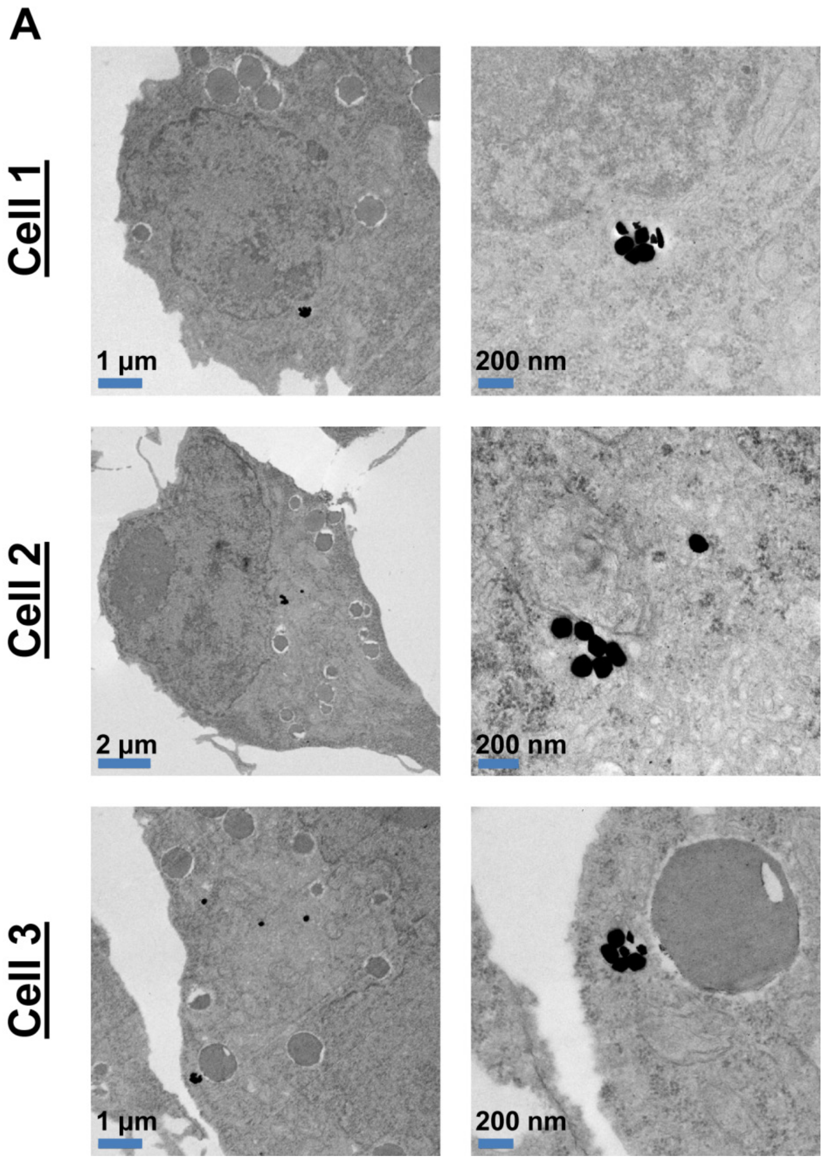

Figure S4 Additional TEM images of Sk-Br-3 cells following 24 h incubation with 24 µg/mL 100 nm PEGylated AuNPs in 0% HuS. TEM images of multiple cells are shown to provide representative sampling of the relative distribution and number of NPs in each cluster (in the two-dimensional slice).

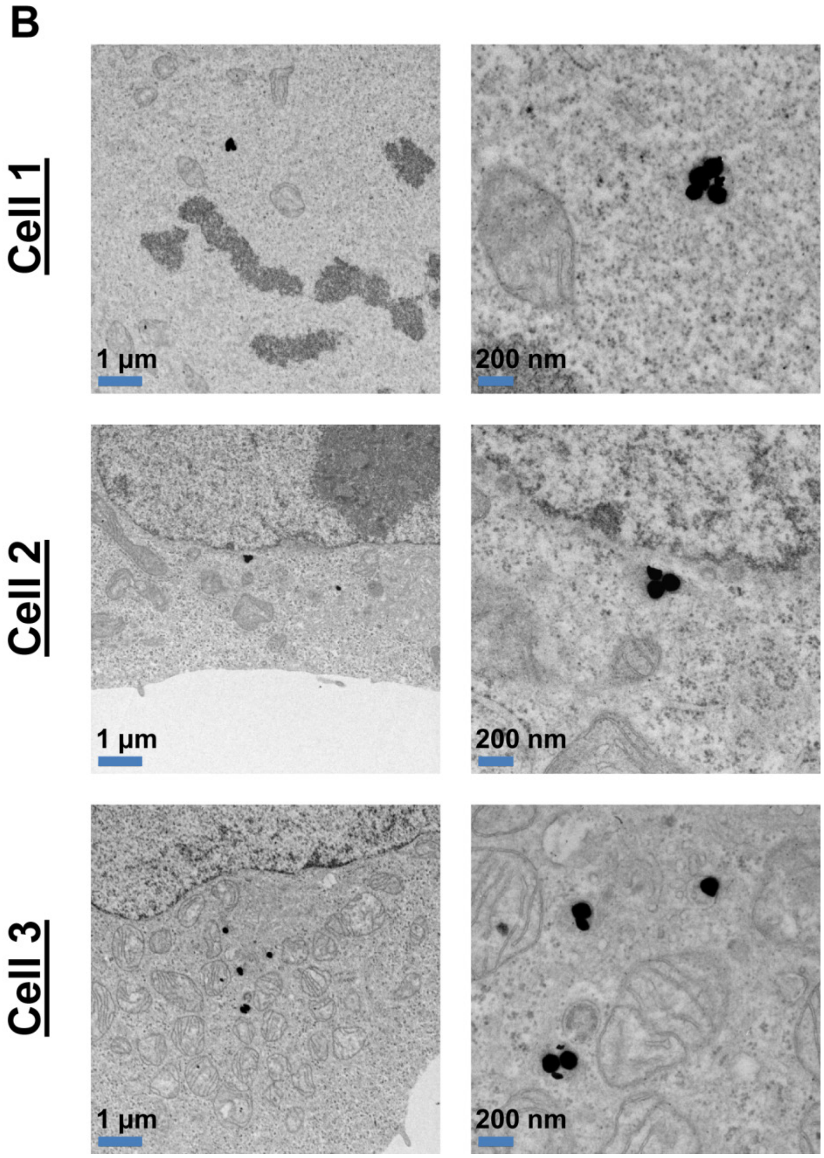

Figure S4 Additional TEM images of Sk-Br-3 cells following 24 h incubation with 24 µg/mL 100 nm PEGylated AuNPs in 10% HuS. TEM images of multiple cells are shown to provide representative sampling of the relative distribution and number of NPs in each cluster (in the two-dimensional slice).

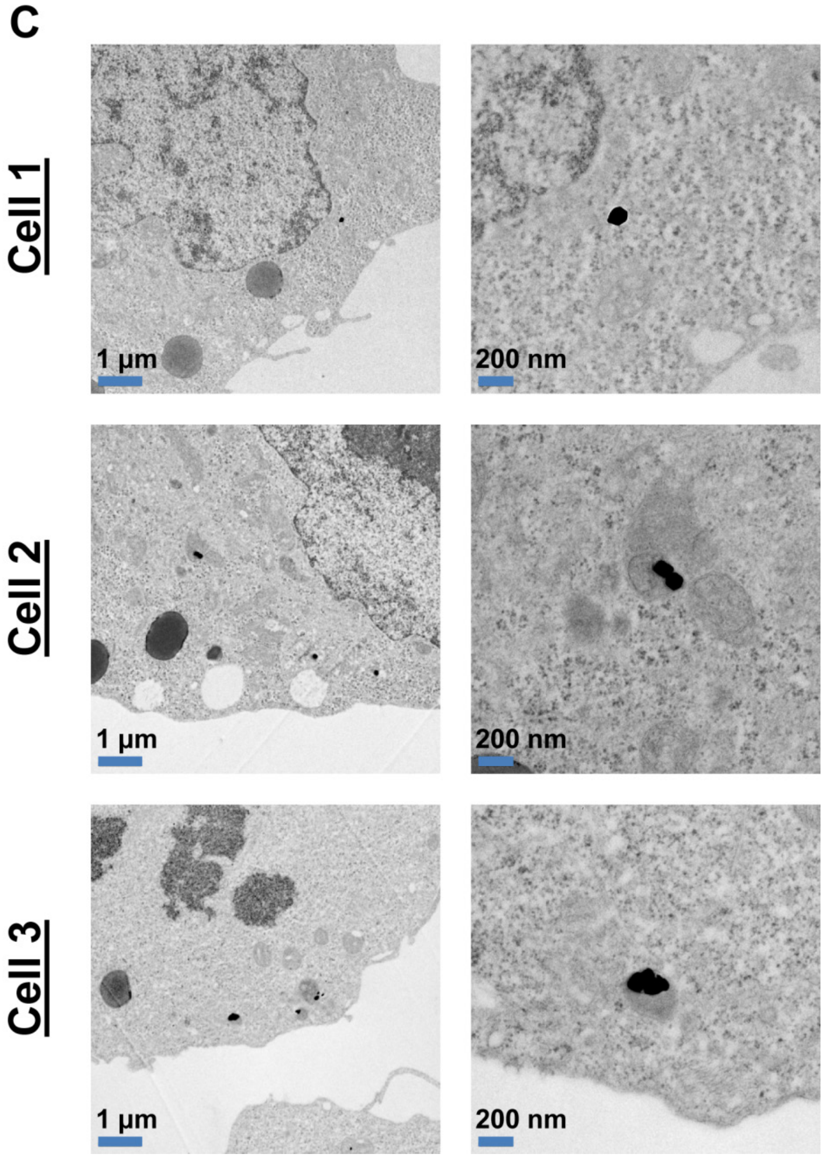

Figure S4 Additional TEM images of Sk-Br-3 cells following 24 h incubation with 24 μg/mL 100 nm PEGylated AuNPs in 25% HuS. TEM images of multiple cells are shown to provide representative sampling of the relative distribution and number of NPs in each cluster (in the two-dimensional slice).

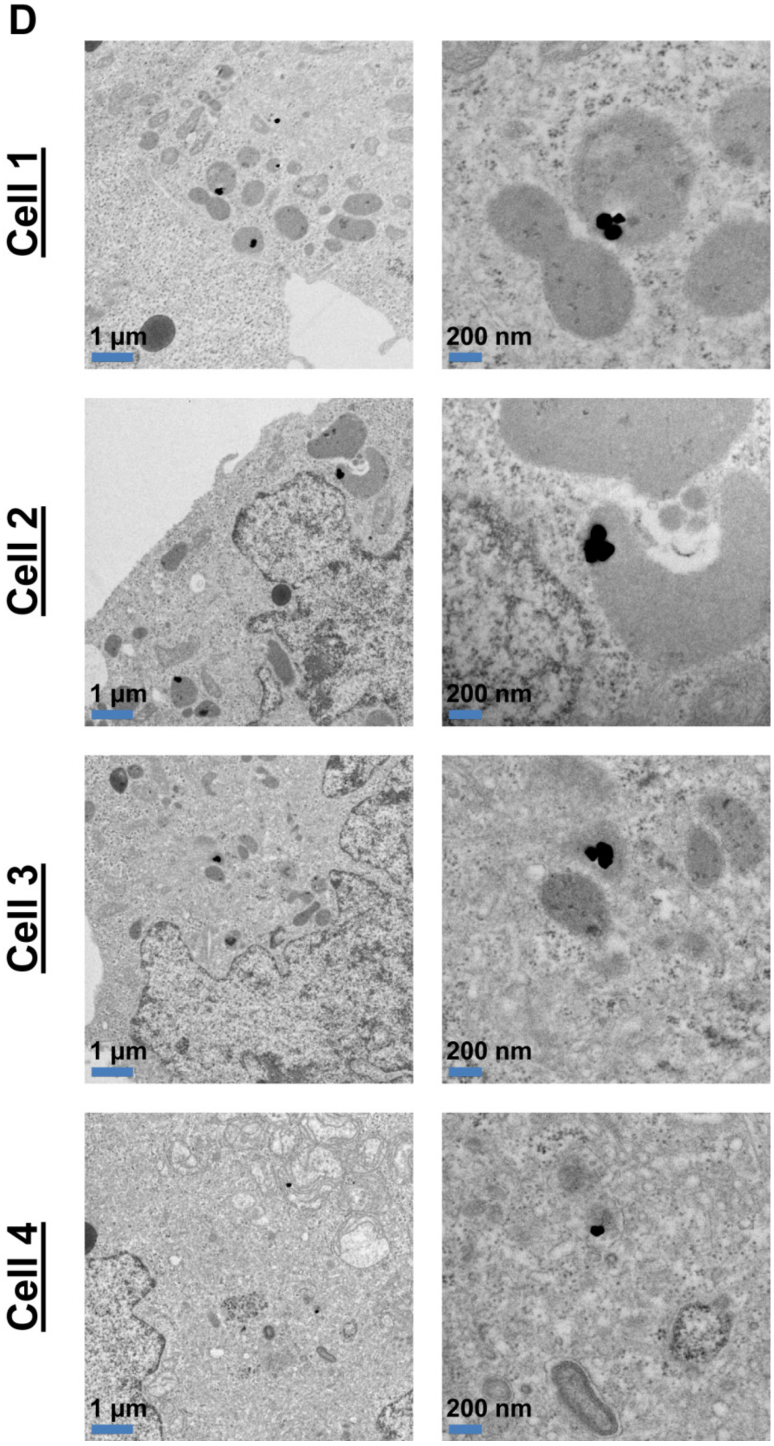

Figure S4 Additional TEM images of Sk-Br-3 cells following 24 h incubation with 24 µg/mL 100 nm PEGylated AuNPs in 50% HuS. TEM images of multiple cells are shown to provide representative sampling of the relative distribution and number of NPs in each cluster (in the two-dimensional slice).
